# Supplementary material for: Genetic and Clinical Profile of Retinopathies Due to Disease-Causing Variants in Leber Congenital Amaurosis (LCA)-Associated Genes in a Large German Cohort
Source: Int J Mol Sci. 2023 May 17;24(10):8915. doi: 10.3390/ijms24108915 (PMC10219005; doi:10.3390/ijms24108915)
Supplement: Supplementary file 1 [file ijms-24-08915-s001.zip › ijms-2363585-supplementary.pdf]

Supplementary Table S1. Patient genotypes and variant classification. The signs “, “”, §, §§, $, $$, *, **, ***, #, ##, °, °°, + mark siblings of one family each.CRD= cone-rod dystrophy; CSNB= congenital stationary night blindness; LCA= Leber congenital amaurosis; LCA_SLS: Senior-Loken syndrome; RP: retinitis pigmentosa.

			Allele 1				***Allele 2***					
Patient ID	**Gene**	**RefSeq transcript**	**cDNA position**	**Amino acid position**	**HGMD ID**	**ACMG classification**	**cDNA position**	**Amino acid position**	**HGMD ID**	**ACMG classification**	**Trans configuration validated**	**Clinical diagnosis**
AIPL1_01	*AIPL1*	NM_014336.5	c.834G>A	p.(Trp278Ter)	CM000003	Pathogenic	c.834G>A	p.(Trp278Ter)	CM000003	Pathogenic	no	LCA
AIPL1_02	*AIPL1*	NM_014336.5	c.834G>A	p.(Trp278Ter)	CM000003	Pathogenic	c.834G>A	p.(Trp278Ter)	CM000003	Pathogenic	no	LCA
AIPL1_03	*AIPL1*	NM_014336.5	c.834G>A	p.(Trp278Ter)	CM000003	Pathogenic	c.834G>A	p.(Trp278Ter)	CM000003	Pathogenic	no	LCA
AIPL1_04	*AIPL1*	NM_014336.5	c.834G>A	p.(Trp278Ter)	CM000003	Pathogenic	c.834G>A	p.(Trp278Ter)	CM000003	Pathogenic	no	LCA
AIPL1_05	*AIPL1*	NM_014336.5	c.834G>A	p.(Trp278Ter)	CM000003	Pathogenic	c.834G>A	p.(Trp278Ter)	CM000003	Pathogenic	no	LCA
AIPL1_06	*AIPL1*	NM_014336.5	c.34dup	p.(Val12GlyfsTer32)	CI2036608	Pathogenic	c.238C>T	p.(Arg80Trp)	CM2036607	Variant of uncertain significance	no	LCA
CABP4_01	*CABP4*	NM_145200.5	c.800_801del	p.(Glu267ValfsTer92)	CM063883	Pathogenic	c.370C>T	p.(Arg124Cys)	CD064514	Likely pathogenic	no	CSNB
CABP4_02	*CABP4*	NM_145200.5	c.646C>T	p.(Arg216Ter)	CM092475	Pathogenic	c.646C>T	p.(Arg216Ter)	CM092475	Pathogenic	no	CSNB
CEP290_01	*CEP290*	NM_025114.4	c.2991+1655A>G	p.(Cys998Ter)	CS064383	Likely pathogenic	c.2991+1655A>G	p.(Cys998Ter)	CS064383	Likely pathogenic	yes	LCA
*CEP290_02**	*CEP290*	NM_025114.4	c.2991+1655A>G	p.(Cys998Ter)	CS064383	Likely pathogenic	c.2991+1655A>G	p.(Cys998Ter)	CS064383	Likely pathogenic	yes	LCA
*CEP290_03**	*CEP290*	NM_025114.4	c.2991+1655A>G	p.(Cys998Ter)	CS064383	Likely pathogenic	c.2991+1655A>G	p.(Cys998Ter)	CS064383	Likely pathogenic	yes	RP
CEP290_04	*CEP290*	NM_025114.4	c.2991+1655A>G	p.(Cys998Ter)	CS064383	Likely pathogenic	c.2991+1655A>G	p.(Cys998Ter)	CS064383	Likely pathogenic	no	LCA
CEP290_05	*CEP290*	NM_025114.4	c.2991+1655A>G	p.(Cys998Ter)	CS064383	Likely pathogenic	c.2991+1655A>G	p.(Cys998Ter)	CS064383	Likely pathogenic	no	LCA
CEP290_06	*CEP290*	NM_025114.4	c.2991+1655A>G	p.(Cys998Ter)	CS064383	Likely pathogenic	c.2991+1655A>G	p.(Cys998Ter)	CS064383	Likely pathogenic	no	LCA
CEP290_07	*CEP290*	NM_025114.4	c.2991+1655A>G	p.(Cys998Ter)	CS064383	Likely pathogenic	c.2991+1655A>G	p.(Cys998Ter)	CS064383	Likely pathogenic	no	RP
CEP290_08	*CEP290*	NM_025114.4	c.2991+1655A>G	p.(Cys998Ter)	CS064383	Likely pathogenic	c.1936C>T	p.(Gln646Ter)	CM071589	Pathogenic	no	RP

CEP290_10	*CEP290*	NM_025114.4	c.2991+1655A>G	p.(Cys998Ter)	CS064383	Likely pathogenic	c.3310-1G>C	p.?	CS075088	Likely pathogenic	yes	LCA
CEP290_11	*CEP290*	NM_025114.4	c.2991+1655A>G	p.(Cys998Ter)	CS064383	Likely pathogenic	c.4882C>T	p.(Gln1628Ter)	CM071586	Pathogenic	yes	RP
CEP290_12	*CEP290*	NM_025114.4	c.2991+1655A>G	p.(Cys998Ter)	CS064383	Likely pathogenic	c.5587-1G>C	p.?	CS072147	Pathogenic	yes	LCA
CEP290_13	*CEP290*	NM_025114.4	c.2991+1655A>G	p.(Cys998Ter)	CS064383	Likely pathogenic	c.5668G>T	p.(Gly1890Ter)	CM061683	Pathogenic	yes	LCA
CEP290_14	*CEP290*	NM_025114.4	c.2991+1655A>G	p.(Cys998Ter)	CS064383	Likely pathogenic	c.5866G>T	p.(Glu1956Ter)	CM063895	Likely pathogenic	no	LCA
CEP290_15	*CEP290*	NM_025114.4	c.2991+1655A>G	p.(Cys998Ter)	CS064383	Likely pathogenic	c.7341dup	p.(Leu2448ThrfsTer8)	CI062252	Pathogenic	no	LCA
CEP290_16	*CEP290*	NM_025114.4	c.2991+1655A>G	p.(Cys998Ter)	CS064383	Likely pathogenic	c.5587-1G>C	p.?	CS072147	Pathogenic	yes	LCA
CEP290_17	*CEP290*	NM_025114.4	c.2991+1655A>G	p.(Cys998Ter)	CS064383	Likely pathogenic	c.3811C>T	p.(Arg1271Ter)	CM072936	Pathogenic	no	RP
CEP290_18	*CEP290*	NM_025114.4	c.2991+1655A>G	p.(Cys998Ter)	CS064383	Likely pathogenic	c.256del	p.(Gln86AsnfsTer2)	CD1920400	Likely pathogenic	no	LCA
CEP290_19	*CEP290*	NM_025114.4	c.2991+1655A>G	p.(Cys998Ter)	CS064383	Likely pathogenic	c.4723A>T	p.(Lys1575Ter)	CM071588	Pathogenic	no	LCA
CEP290_20#	*CEP290*	NM_025114.4	c.2991+1655A>G	p.(Cys998Ter)	CS064383	Likely pathogenic	c.6964A>T	p.(Lys2322Ter)	CM140480	Likely pathogenic	yes	LCA
CEP290_21#	*CEP290*	NM_025114.4	c.2991+1655A>G	p.(Cys998Ter)	CS064383	Likely pathogenic	c.6964A>T	p.(Lys2322Ter)	CM140480	Likely pathogenic	yes	RP
CEP290_22	*CEP290*	NM_025114.4	c.5493del	p.(Ala1832ProfsTer19)	CD073587	Pathogenic	c.5587-1G>C	p.?	CS072147	Pathogenic	no	LCA
CEP290_23	*CEP290*	NM_025114.4	c.4029+1G>A	p.?	CS1920401	Pathogenic	c.7048C>T	p.(Gln2350Ter)	CM1723409	Pathogenic	yes	RP
CRB1_01§	*CRB1*	NM_201253.3	c.3299T>G	p.(Ile1100Arg)	CM012917	Likely pathogenic	c.3331G>T	p.(Glu1111Ter)	CM012918	Likely pathogenic	no	LCA
CRB1_02§	*CRB1*	NM_201253.3	c.3299T>G	p.(Ile1100Arg)	CM012917	Likely pathogenic	c.3331G>T	p.(Glu1111Ter)	CM012918	Likely pathogenic	no	LCA
CRB1_03°	*CRB1*	NM_201253.3	c.2234C>T	p.(Thr745Met)	CM992150	Pathogenic	c.2234C>T	p.(Thr745Met)	CM992150	Pathogenic	no	RP
CRB1_04°	*CRB1*	NM_201253.3	c.2234C>T	p.(Thr745Met)	CM992150	Pathogenic	c.2234C>T	p.(Thr745Met)	CM992150	Pathogenic	no	RP
CRB1_05	*CRB1*	NM_201253.3	c.1171+2T>G	p.?	CS2036665	Likely pathogenic	c.1171+2T>G	p.?	CS2036665	Likely pathogenic	no	RP
CRB1_06+	*CRB1*	NM_201253.3	c.2843G>A	p.(Cys948Tyr)	CM992152	Pathogenic	c.2843G>A	p.(Cys948Tyr)	CM992152	Pathogenic	yes	RP
CRB1_07$	*CRB1*	NM_201253.3	c.2843G>A	p.(Cys948Tyr)	CM992152	Pathogenic	c.2843G>A	p.(Cys948Tyr)	CM992152	Pathogenic	yes	LCA
CRB1_08$	*CRB1*	NM_201253.3	c.2843G>A	p.(Cys948Tyr)	CM992152	Pathogenic	c.2843G>A	p.(Cys948Tyr)	CM992152	Pathogenic	yes	LCA
CRB1_09+	*CRB1*	NM_201253.3	c.2843G>A	p.(Cys948Tyr)	CM992152	Pathogenic	c.2843G>A	p.(Cys948Tyr)	CM992152	Pathogenic	yes	RP
CRB1_10	*CRB1*	NM_201253.3	c.1023T>A	p.(Asn341Lys)	-	Variant of uncertain significance	c.3713_3716dup	p.(Cys1240ProfsTer24)	CI106997	Pathogenic	no	RP
CRB1_11	*CRB1*	NM_201253.3	c.2230C>T	p.(Arg744Ter)	BM1462770	Pathogenic	c.2230C>T	p.(Arg744Ter)	BM1462770	Pathogenic	yes	LCA
CRB1_12	*CRB1*	NM_201253.3	c.2230C>T	p.(Arg744Ter)	BM1462770	Pathogenic	c.2230C>T	p.(Arg744Ter)	BM1462770	Pathogenic	yes	RP
CRB1_14	*CRB1*	NM_201253.3	c.1483del	p.(Trp495GlyfsTer7)	CD2036666	Pathogenic	c.3934T>A	p.(Cys1312Ser)	CM2036672	Likely pathogenic	yes	RP

CRB1_15	*CRB1*	NM_201253.3	c.1181G>A	p.(Cys394Tyr)	-	Likely pathogenic	c.1181G>A	p.(Cys394Tyr)	-	Likely pathogenic	no	LCA
CRB1_16	*CRB1*	NM_201253.3	c.803_806del	p.(Ser268AsnfsTer33)	CD2036673	Pathogenic	c.2234C>T	p.(Thr745Met)	CM992150	Pathogenic	no	RP
*CRB1_17***	*CRB1*	NM_201253.3	c.407G>A	p.(Cys136Tyr)	CM160492	Pathogenic	c.1465G>T	p.(Glu489Ter)	CM160493	Likely pathogenic	yes	CRD
*CRB1_18***	*CRB1*	NM_201253.3	c.407G>A	p.(Cys136Tyr)	CM160492	Pathogenic	c.1465G>T	p.(Glu489Ter)	CM160493	Likely pathogenic	yes	RP
CRB1_19	*CRB1*	NM_201253.3	c.2308G>A	p.(Gly770Ser)	CM165114	Pathogenic	c.2308G>A	p.(Gly770Ser)	CM165114	Pathogenic	no	RP
CRB1_20	*CRB1*	NM_201253.3	c.2248G>A	p.(Gly750Ser)	CM140495	Pathogenic	c.2248G>A	p.(Gly750Ser)	CM140495	Pathogenic	yes	RP
CRB1_21	*CRB1*	NM_201253.3	c.1349G>A	p.(Cys450Tyr)	CM2123993	Pathogenic	c.3541T>C	p.(Cys1181Arg)	CM012919	Likely pathogenic	no	RP
CRB1_22"	*CRB1*	NM_201253.3	c.506del	p.(Gly169ValfsTer37)	CD140516	Pathogenic	c.2290C>T	p.(Arg764Cys)	CM992151	Pathogenic	no	RP
CRB1_23"	*CRB1*	NM_201253.3	c.506del	p.(Gly169ValfsTer37)	CD140516	Pathogenic	c.2290C>T	p.(Arg764Cys)	CM992151	Pathogenic	no	RP
CRX_01	*CRX*	NM_000554.6	c.128G>A	p.(Arg43His)	CM1920491	Pathogenic	c.128G>A	p.(Arg43His)	CM1920491	Pathogenic	no	LCA
IFT140_01	*IFT140*	NM_014714.4	c.472C>T	p.(Arg158Trp)	CM160497	Variant of uncertain significance	c.1565G>A	p.(Gly522Gu)	CM123479	Pathogenic	no	RP
*IQCB1_01****	*IQCB1*	NM_001023570.4	c.1558C>T	p.(Gln520Ter)	CM160496	Pathogenic	c.1558C>T	p.(Gln520Ter)	CM160496	Pathogenic	yes	LCA
*IQCB1_02****	*IQCB1*	NM_001023570.4	c.1558C>T	p.(Gln520Ter)	CM160496	Pathogenic	c.1558C>T	p.(Gln520Ter)	CM160496	Pathogenic	yes	LCA
*IQCB1_03****	*IQCB1*	NM_001023570.4	c.1558C>T	p.(Gln520Ter)	CM160496	Pathogenic	c.1558C>T	p.(Gln520Ter)	CM160496	Pathogenic	yes	LCA
IQCB1_04	*IQCB1*	NM_001023570.4	c.424_425del	p.(Phe142ProfsTer5)	CD050884	Pathogenic	c.424_425del	p.(Phe142ProfsTer5)	CD050884	Pathogenic	no	LCA
IQCB1_05	*IQCB1*	NM_001023570.4	c.825_828del	p.(Arg275SerfsTer6)	CD050886	Pathogenic	c.1518_1519del	p.(His506GlnfsTer13)	CD050887	Pathogenic	no	LCA_SLS
LCA5_01	*LCA5*	NM_181714.4	c.516_519del	p.(Lys172AsnfsTer3)	CD2036712	Pathogenic	c.401A>C	p.(Lys134Thr)	CM2036711	Variant of uncertain significance	no	RP
LRAT_01	*LRAT*	NM_004744.5	c.449dup	p.(Phe151LeufsTer33)	CI1312840	Likely pathogenic	c.449dup	p.(Phe151LeufsTer33)	CI1312840	Likely pathogenic	no	RP
LRAT_02##	*LRAT*	NM_004744.5	c.481T>C	p.(Cys161Arg)	-	Variant of uncertain significance	c.481T>C	p.(Cys161Arg)	-	Variant of uncertain significance	yes	RP
LRAT_03##	*LRAT*	NM_004744.5	c.481T>C	p.(Cys161Arg)	-	Variant of uncertain significance	c.481T>C	p.(Cys161Arg)	-	Variant of uncertain significance	yes	RP
NMNAT1_01	*NMNAT1*	NM_022787.4	c.629T>C	p.(Ile210Thr)	CM2036723	Likely pathogenic	c.769G>A	p.(Glu257Lys)	CM127755	Pathogenic	no	LCA

NMNAT1_02	*NMNAT 1*	NM_022787.4	c.253T>C	p.(Trp85Arg)	CM145932	Likely pathogenic	c.769G>A	p.(Glu257Lys)	CM127755	Pathogenic	no	LCA
RD3_01	*RD3*	NM_183059.3	c.112C>T	p.(Arg38Ter)	CM130361	Pathogenic	c.112C>T	p.(Arg38Ter)	CM130361	Pathogenic	no	LCA
RDH12_01	*RDH12*	NM_152443.3	c.451C>G	p.(His151Asp)	CM042468	Pathogenic	c.464C>T	p.(Thr155Ile)	CM054828	Pathogenic	no	RP
RDH12_02	*RDH12*	NM_152443.3	c.146C>T	p.(Thr49Met)	CM042103	Pathogenic	c.451C>G	p.(His151Asp)	CM042468	Pathogenic	no	LCA
RDH12_03	*RDH12*	NM_152443.3	c.464C>T	p.(Thr155Ile)	CM054828	Pathogenic	c.806_810del	p.(Ala269GlyfsTer2)	CD042224	Pathogenic	no	LCA
RDH12_04	*RDH12*	NM_152443.3	c.806_810del	p.(Ala269GlyfsTer2)	CD042224	Pathogenic	c.806_810del	p.(Ala269GlyfsTer2)	CD042224	Pathogenic	no	RP
RDH12_05	*RDH12*	NM_152443.3	c.806_810del	p.(Ala269GlyfsTer2)	CD042224	Pathogenic	c.806_810del	p.(Ala269GlyfsTer2)	CD042224	Pathogenic	no	LCA
RDH12_06	*RDH12*	NM_152443.3	c.806_810del	p.(Ala269GlyfsTer2)	CD042224	Pathogenic	c.451C>G	p.(His151Asp)	CM042468	Pathogenic	no	RP
RDH12_07	*RDH12*	NM_152443.3	c.464C>T	p.(Thr155Ile)	CM054828	Pathogenic	c.464C>T	p.(Thr155Ile)	CM054828	Pathogenic	no	RP
RDH12_08	*RDH12*	NM_152443.3	c.481C>T	p.(Arg161Trp)	CM118733	Likely pathogenic	c.632C>T	p.(Thr211Ile)	-	Variant of uncertain significance	no	RP
RDH12_09	*RDH12*	NM_152443.3	c.444C>A	p.(His148Gln)	-	Likely pathogenic	c.444C>A	p.(His148Gln)	-	Likely pathogenic	no	RP
RDH12_10	*RDH12*	NM_152443.3	c.379G>T	p.(Gly127Ter)	CM042466	Pathogenic	c.379G>T	p.(Gly127Ter)	CM042466	Pathogenic	no	RP
RDH12_11	*RDH12*	NM_152443.3	c.565C>T	p.(Gln189Ter)	CM042105	Pathogenic	c.565C>T	p.(Gln189Ter)	CM042105	Pathogenic	no	LCA
RDH12_12	*RDH12*	NM_152443.3	c.448+1G>C	p.?	CS2040299	Pathogenic	c.609C>A	p.(Ser203Arg)	CM118730	Pathogenic	no	RP
RDH12_13	*RDH12*	NM_152443.3	c.821T>C	p.(Leu274Pro)	CM054835	Likely pathogenic	c.821T>C	p.(Leu274Pro)	CM054835	Likely pathogenic	yes	RP
RDH12_14	*RDH12*	NM_152443.3	c.599A>C	p.(Tyr200Ser)	CM2036764	Pathogenic	c.599A>C	p.(Tyr200Ser)	CM2036764	Pathogenic	no	RP
RPE65_01§§	*RPE65*	NM_000329.3	c.1451G>T	p.(Gly484Val)	CM2027966	Pathogenic	c.1451G>T	p.(Gly484Val)	CM2027966	Pathogenic	yes	LCA
RPE65_02§§	*RPE65*	NM_000329.3	c.1451G>T	p.(Gly484Val)	CM2027966	Pathogenic	c.1451G>T	p.(Gly484Val)	CM2027966	Pathogenic	yes	LCA
RPE65_03§§	*RPE65*	NM_000329.3	c.1451G>T	p.(Gly484Val)	CM2027966	Pathogenic	c.1451G>T	p.(Gly484Val)	CM2027966	Pathogenic	yes	LCA
RPE65_04§§	*RPE65*	NM_000329.3	c.1451G>T	p.(Gly484Val)	CM2027966	Pathogenic	c.1451G>T	p.(Gly484Val)	CM2027966	Pathogenic	yes	LCA
RPE65_05§§	*RPE65*	NM_000329.3	c.1451G>T	p.(Gly484Val)	CM2027966	Pathogenic	c.1451G>T	p.(Gly484Val)	CM2027966	Pathogenic	yes	LCA
RPE65_06	*RPE65*	NM_000329.3	c.11+5G>A	p.?	CS971898	Pathogenic	c.130C>T	p.(Arg44Ter)	CM167086	Pathogenic	no	LCA
RPE65_07	*RPE65*	NM_000329.3	c.11+5G>A	p.?	CS971898	Pathogenic	c.1102T>C	p.(Tyr368His)	CM021674	Pathogenic	no	RP
RPE65_08	*RPE65*	NM_000329.3	c.11+5G>A	p.?	CS971898	Pathogenic	c.725+2T>A	p.?	CS128120	Pathogenic	no	LCA
RPE65_09	*RPE65*	NM_000329.3	c.272G>A	p.(Arg91Gln)	CM003832	Pathogenic	c.825C>G	p.(Tyr275Ter)	CM1821771	Pathogenic	yes	LCA
RPE65_10	*RPE65*	NM_000329.3	c.271C>T	p.(Arg91Trp)	CM983758	Pathogenic	c.271C>T	p.(Arg91Trp)	CM983758	Pathogenic	no	LCA
RPE65_11	*RPE65*	NM_000329.3	c.304G>T	p.(Glu102Ter)	CM994069	Pathogenic	c.304G>T	p.(Glu102Ter)	CM994069	Pathogenic	no	LCA
RPE65_12	*RPE65*	NM_000329.3	c.1543C>T	p.(Arg515Trp)	CM045193	Pathogenic	c.1543C>T	p.(Arg515Trp)	CM045193	Pathogenic	no	RP

RPE65_13	*RPE65*	NM_000329.3	c.283G>C	p.(Glu95Gln)	CM005351	Likely pathogenic	c.283G>C	p.(Glu95Gln)	CM005351	Likely pathogenic	yes	RP
RPE65_14	*RPE65*	NM_000329.3	c.130C>T	p.(Arg44Ter)	CM167086	Pathogenic	c.1102T>C	p.(Tyr368His)	CM021674	Pathogenic	no	LCA
RPE65_15	*RPE65*	NM_000329.3	c.1102T>C	p.(Tyr368His)	CM021674	Pathogenic	c.1102T>C	p.(Tyr368His)	CM021674	Pathogenic	yes	LCA
RPGRIP1_01	*RPGRIP1*	NM_020366.4	c.2021C>A	p.(Pro674His)	CM156188	Variant of uncertain significance	c.2021C>A	p.(Pro674His)	CM156188	Variant of uncertain significance	yes	LCA
RPGRIP1_02	*RPGRIP1*	NM_020366.4	c.2432T>A	p.(Leu811His)	CM2036785	Variant of uncertain significance	c.3239_3339del	p.?	CG2034439	Likely pathogenic	no	CRD
SPATA7_01$$	*SPATA7*	NM_018418.5	c.1112T>C	p.(Ile371Thr)	CM1312877	Likely pathogenic	c.1112T>C	p.(Ile371Thr)	CM1312877	Likely pathogenic	yes	RP
SPATA7_02$$	*SPATA7*	NM_018418.5	c.1112T>C	p.(Ile371Thr)	CM1312877	Likely pathogenic	c.1112T>C	p.(Ile371Thr)	CM1312877	Likely pathogenic	yes	CRD
TULP1_1 °°	*TULP1*	NM_003322.6	c.1081C>T	p.(Arg361Ter)	CM140477	Likely pathogenic	c.1258C>A	p.(Arg420Ser)	CM135101	Likely pathogenic	yes	RP
TULP1_2 °°	*TULP1*	NM_003322.6	c.1081C>T	p.(Arg361Ter)	CM140477	Likely pathogenic	c.1258C>A	p.(Arg420Ser)	CM135101	Likely pathogenic	yes	RP
TULP1_3 ""	*TULP1*	NM_003322.6	c.1025G>A	p.(Arg342Gln)	CM119412	Likely pathogenic	c.1496-6C>A	p.(Pro499LeufsTer143)	CS984713	Pathogenic	yes	CRD
TULP1_4 ""	*TULP1*	NM_003322.6	c.1025G>A	p.(Arg342Gln)	CM119412	Likely pathogenic	c.1496-6C>A	p.(Pro499LeufsTer143)	CS984713	Pathogenic	yes	CRD
TULP1_5	*TULP1*	NM_003322.6	c.629C>G	p.(Ser210Ter)	CM140491	Pathogenic	c.629C>G	p.(Ser210Ter)	CM140491	Pathogenic	no	LCA
TULP1_6	*TULP1*	NM_003322.6	c.1199G>A	p.(Arg400Gln)	CM095222	Pathogenic	c.1268T>C	p.(Val423Ala)	CM2036798	Pathogenic	yes	RP
